# Supplementary material for: Mathematical modelling of bicarbonate supplementation and acid-base chemistry in kidney failure patients on hemodialysis
Source: PLoS One. 2023 Feb 24;18(2):e0282104. doi: 10.1371/journal.pone.0282104 (PMC9955675; doi:10.1371/journal.pone.0282104)
Supplement: S1 Text — Equations of the model (appendices A, B, C, D) and results of the sensitivity analysis (appendix E). (PDF) [file pone.0282104.s001.pdf]

Supporting Information for:

"Mathematical modelling of bicarbonate supplementation and acid-base chemistry in kidney failure patients on hemodialysis"

Mauro Pietribiasi, Jacek Waniewski, and John K. Leypoldt

Appendices

Appendix A

Acid-base status equations for blood capillaries

The following system of equations is described in detail in a previously published article [1], and here is given only a condensed formulation. Supplementary Equations A1 – A20 can be solved simultaneously given a combination of known variables obtained from the fixed parameters of the model (Table 1 in the main text) and solution of the state equations.

Symbols

- tCO_2 – total CO_2 concentration (mmol/L).
- tO_2 – total oxygen concentration (mmol/L).
- pCO_2 – CO_2 partial pressure (kPa).
- pO_2 – oxygen partial pressure (kPa).
- *Bic* – bicarbonate concentration (mmol/L).
- *Hct* – haematocrit.
- σ_{CO2}, σ_{O2} – CO_2, O_2 solubility (mmol/L/kPa).
- $HBNHCO_2^-$, $HbO_2NHCO_2^-$, $HbNH_3^+$, $HbO_2NH_3^+$, $HbNH_2$, HbO_2NH_2 - molecular variants of hemoglobin amino end (mmol/L).
- $Hb(RH)_b$, $Hb(R^-)_b$, $HbO_2(RH)_b$, $HbO_2(R^-)_b$ - molecular variants of hemoglobin side chains (mmol/L).
- Hb_e – total hemoglobin concentration (mmol/L).
- sO_2 – oxygen saturation.
- Ψ_{37° - oxygen saturation at 37C, as a function of pH_p, pO_2, and pCO_2 [2].
- $HNBBp$, NBB_p^- - molecular variants of non-bicarbonate buffer base (mEq/L).
- *BE* – base excess (mmol/L)
- *nBB* – total buffer base under standard conditions (mmol/L) [3].
- *pK* – acid dissociation constant.

Subscripts

- *p* – plasma
- *e* – erythrocytes

Equations

$$tCO_2 = \left(\sigma_{CO2,p} pCO_2 + Bic_p\right)\left(1 - Hct\right) + \left(\sigma_{CO2,e} pCO_2 + Bic_e + HbNHCO_2^- + HbO_2NHCO_2^-\right)Hct \quad \text{(A1)}$$

$$tO_2 = \sigma_{O2,p} pO_2 \left(1 - Hct\right) + \left(\sigma_{O2,e} pO_2 + sO_2 Hb_e\right)Hct \quad \text{(A2)}$$

$$Hb_e = HbNH_3^+ + HbNH_2 + HbNHCO_2^- + HbO_2NH_3^+ + HbO_2NH_2 + HbO_2NHCO_2^- \quad \text{(A3)}$$

$$Hb_e = Hb(RH)_b + Hb(R^-)_b + HbO_2(RH)_b + HbO_2(R^-)_b \quad \text{(A4)}$$

$$sO_2 = \left(HbO_2NH_3^+ + HbO_2NH_2 + HbO_2NHCO_2^-\right) / Hb_e \quad \text{(A5)}$$

$$sO_2 = \left(HbO_2(RH)_b + HbO_2(R^-)_b\right) / Hb_e \quad \text{(A6)}$$

$$sO_2 = \Psi_{37^\circ}\left(pH_p, pO_2, pCO_2\right) \quad \text{(A7)}$$

$$pH_e = 7.19 + 0.77(pH_p - 7.4) + 0.031\left(1 - sO_2\right) \quad \text{(A8)}$$

$$tNBB_p = HNBB_p + NBB^-{}_p \quad \text{(A9)}$$

$$\begin{aligned} BE &= \left(Bic_p + NBB_p^-\right)\left(1 - Hct\right) \\ &+ \left[Bic_e + b\left(Hb(R^-)_b\right) + b\left(HbO_2(R^-)_b\right) + HbNH_2 + HbO_2NH_2\right]Hct \\ &+ \left[2\left(HbNHCO_2^-\right) + 2\left(HbO_2NHCO_2^-\right)\right]Hct - nBB \end{aligned} \quad \text{(A10)}$$

$$nBB = 41.7\left(1 - Hct\right) + 72.5 \cdot Hct \;\; [3] \quad \text{(A11)}$$

Two mass action equations for plasma pH and seven for erythrocytes pH are written, one for each pair of weak acid *HA* and base A^- (CO_2 and bicarbonate, non-bicarbonate buffer and hemoglobin dissociated forms):

$$pH = pK_A + \log\left(\frac{A^-}{HA}\right) \quad \text{(A12)- (A20)}$$

Appendix B

Acid-base status equations for interstitial fluid and tissue cells

Supplementary Equations A21 – A30 are solved simultaneously to give the value of variables describing the interstitial fluid and tissue cells acid-base status. The system is described in detail in another paper [4].

Symbols

V – volume (L).
ϕ_{pH} – sensitivity of intracellular pH to changes in interstitial pH.
β_{NBBt} – buffer capacity of non-bicarbonate tissue buffer (mmol/L/pH).
nBB_i – normal total interstitial buffer base [4]
npH_i – normal interstitial pH [4]
$nBic_t$ – normal tissue cells bicarbonate concentration [4]
npH_t – normal tissue cells pH [4]

Subscripts

it – average of interstitial fluid and tissue cells.
i – interstitial.
t – tissue cells.

Equations

$$tCO_{2,it} = \frac{\left(Bic_i + \sigma_{CO2,i} pCO_{2,i}\right)V_i + \left(Bic_t + \sigma_{CO2,t} pCO_{2,t}\right)V_t}{V_i + V_t} \tag{A21}$$

$$pCO_{2,i} = pCO_{2,t} \tag{A22}$$

$$tNBB_i = HNBB_i + NBB_i^- \tag{A23}$$

$$BE_{it} = \frac{BE_i V_i + BE_t V_t}{V_i + V_t} \tag{A24}$$

$$BE_i = Bic_i + NBB_i^- - nBB_i \tag{A25}$$

$$BE_t = Bic_t - nBic_t + \beta_{NBBt}\left(pH_t - npH_t\right) \tag{A26}$$

$$pH_i = pK_{Bic,i} + \log\left(\frac{Bic_i}{\sigma_{CO2,i} pCO_{2,i}}\right) \tag{A27}$$

$$pH_i = pK_{NBB,i} + \log\left(\frac{NBB_i^-}{HNBB_i}\right) \tag{A28}$$

$$pH_t = pK_{Bic,t} + \log\left(\frac{Bic_t}{\sigma_{Bic,t} pCO_{2,t}}\right) \quad \text{(A29)}$$

$$pH_t = npH_t + \phi_{pH} \cdot \left(pH_i - npH_i\right) \quad \text{(A30)}$$

Appendix C

Solution of the pre-dialysis steady-state model

Clinical values of arterial plasma bicarbonate concentration and pCO_2 were provided in the studies by Sargent et al and Park et al [5, 6]. To complete the initial input to the model a third variable is needed [4]: for lack of data, the value of arterial oxygen saturation was assumed equal to 0.97; $sO_{2,a}$ was chosen because it is easily measurable in a clinical setting; however, any other variable describing the acid-base status of blood (Appendix C) could be used. From these three inputs the system of equations describing the arterial blood biochemistry was solved univocally. One difference from the original model was the assumption that the pulmonary shunt flow was negligible; from this follows that the quantities in the lung capillary (subscript *l*) are equal to those in arterial blood (subscript *a*):

$$\begin{aligned} tCO_{2,l} &= tCO_{2,a} \\ tO_{2,l} &= tO_{2,a} \\ BE_l &= BE_a \end{aligned} \quad \text{(A31)}$$

where tCO_2 , tO_2, and BE are total CO_2, total oxygen and base excess, respectively. The three quantities thus calculated were used to solve the equations for the lung compartment, and obtain the partial pressures $pCO_{2,L}$ and $pO_{2,L}$, from which the fractions of the expired CO_2 ($FeCO_2$) and oxygen (FeO_2) were calculated:

$$FeCO_2 = \frac{pCO_{2,l}}{p_b - pH2O} \quad \text{(A32)}$$

$$FeO_2 = \frac{pO_{2,l}}{p_b - p_{H20}} \quad \text{(A33)}$$

Parameters p_b and p_{H20} are the barometric pressure and the pressure of saturated water vapor at 37 C, respectively.

Before the start of dialysis, the gas flows between environment and lungs, $\dot{V}CO_{2,Ae}$ and $\dot{V}O_{2,eA}$, are assumed to be in equilibrium with the tissue production of CO_2 ($\dot{V}CO_{2,t}$) and consumption of O_2 ($\dot{V}O_{2,t}$), respectively:

$$\dot{V}CO_{2,Ae} = \dot{V}CO_{2,t} = \dot{V}_E \left(FeCO_2 - FiCO_2 \right) \tag{A34}$$

$$\dot{V}O_{2,eA} = \dot{V}O_{2,t} = \dot{V}_E \left(FiO_2 - FeO_2 \right) \tag{A35}$$

$$\dot{V}_E = f \left(V_T - V_D \right) \tag{A36}$$

where $\dot{V}_E$ is the minute ventilation, f the respiration rate, V_T the pulmonary tidal volume, and V_D the dead volume. Tidal volume was calculated as a fraction of body weight, assuming a physiological normalized tidal volume of 8 ml/Kg body weight; dead volume was considered equal to one third of tidal volume. $FiCO_2$ and FiO_2 are constants representing the fractions of inspired CO_2 and O_2, respectively. It follows that:

$$\dot{V}_E = \frac{\dot{V}CO_{2,t}}{\left(FeCO_2 - FiCO_2 \right)} \tag{A37}$$

$$\dot{V}_E = \frac{\dot{V}O_{2,t}}{\left(FiO_2 - FeO_2 \right)} \tag{A38}$$

The values of $\dot{V}CO_{2,t}$ and $\dot{V}O_{2,t}$ were estimated via least square optimization so that, given the input bicarbonate, $pCO_{2,a}$ and $sO_{2,a}$, the values of $\dot{V}_E$ calculated from Equations A37 and A38 would be equal. The two parameters thus identified are then treated as constants for the simulation of the HD session. The remaining values necessary to calculate the initial, pre-dialysis state of the model, are obtained simply imposing the steady state of the state equations as described in Appendix B and reference [4].

Appendix D

Time-dependent equations of the model

The state equations of the model are given in a condensed form, the detailed description can be found in reference [4].

Symbols

- $FeCO_2$, FeO_2 – expired fractions of CO_2, O_2.
- $FiCO_2$, FiO_2 – inspired fractions of CO_2, O_2.
- $\dot{Q}_{CO}$ – cardiac output (L/min).
- $\dot{V}_E$ – minute ventilation (L/min).
- V – volume (L).
- tCO_2, tO_2 – total carbon dioxide, total oxygen (mmol/L).
- BE – base excess (mmol/L).
- $\dot{V}CO_{2,t}$ - tissue production of CO_2.
- H_t – net acid production rate (mmol/min).

Subscripts

- *A – lung alveoli.*
- *l – lung capillary.*
- *a – arterial.*
- *mv – mixed-venous.*
- *v – venous.*

Lung alveoli and capillaries equations

$$\begin{aligned}\frac{d}{dt}\left(FeCO_2\right) &= \frac{\dot{Q}_{CO}\left(tCO_{2,mv}-tCO_{2,l}\right)-\dot{V}_E\left(FeCO_2-FiCO_2\right)}{V_A}\\ \frac{d}{dt}\left(FeO_2\right) &= \frac{\dot{V}_E\left(FiO_2-FeO_2\right)-\dot{Q}_{CO}\left(tO_{2,l}-tO_{2,mv}\right)}{V_A}\end{aligned} \qquad \text{(A39)}$$

$$\begin{aligned}pCO_{2,l} &= pCO_{2,A} = FeCO_2\left(p_b-p_{H2O}\right)\\ pO_{2,l} &= pO_{2,A} = FeO_2\left(p_b-p_{H2O}\right)\end{aligned} \qquad \text{(A40)}$$

$$BE_l = BE_{mv} \qquad \text{(A41)}$$

BE_l, $pCO_{2,l}$ and $pO_{2,l}$ are used to solve the system for blood in the lung capillaries (Appendix C), calculating $tCO_{2,l}$ and $tO_{2,l}$.

Arterial capillaries equations

$$\begin{aligned}
&\frac{d}{dt}\left(tCO_{2,a}\right)=\frac{\dot{Q}_{CO}\left(tCO_{2,l}-tCO_{2,a}\right)}{V_a}\\
&\frac{d}{dt}\left(tO_{2,a}\right)=\frac{\dot{Q}_{CO}\left(tO_{2,l}-tO_{2,a}\right)}{V_a}\\
&\frac{d}{dt}\left(BE_a\right)=\frac{\dot{Q}_{CO}\left(BE_l-BE_a\right)}{V_a}
\end{aligned} \tag{A42}$$

Tissue capillaries, interstitial fluid and tissue cells equations

$tCO_{2,it}$ and BE_{it} are used to solve the system for interstitial fluid and tissue cells (Appendix D), calculating $pCO_{2,i}=pCO_{2,v}$ and $Bic_i=Bic_v$ where the subscript *v* indicates venous blood immediately after the exchange with the tissues. These, together with $tO_{2,v}=tO_{2,a}-\dot{Q}_{CO}^{-1}\left(\dot{V}O_{2,t}\right)$ are used to solve the system (Appendix C) for the venous capillaries, giving $tCO_{2,v}$ and BE_v.

$$\begin{aligned}
&\frac{d}{dt}\left(tCO_{2,it}\right)=\frac{\dot{V}CO_{2,t}-\dot{Q}_{CO}\left(tCO_{2,v}-tCO_{2,a}\right)}{V_i+V_t}\\
&\frac{d}{dt}\left(BE_{it}\right)=\frac{-H_t-\dot{Q}_{CO}\left(BE_v-BE_a\right)}{V_i+V_t}
\end{aligned} \tag{A43}$$

Post-dialyzer blood and mixed-venous capillaries equations

$tCO_{2,a}$, $tO_{2,a}$ and BE_a are used to solve the system for arterial capillaries (Appendix C), to calculated Bic_a and $pCO_{2,a}$ used in the Equations 1 – 3 in the main text to calculate the composition of the blood entering the mixed-venous compartment, whose state equations were shown in Equation 4.

Appendix E

Sensitivity Analysis

Local sensitivity analysis of the model was carried out for the parameters reported in Table 1 in the main text and the dialysances of bicarbonate and dissolved CO_2, using a OAT (one-at-a-time) approach, that is perturbing only parameter and calculating the difference in the output. The sensitivity indices $S_{i,j}$ were calculated as the partial derivative of the i-th output Y_i with respect to the j-th input parameter P_j, approximated with a finite difference: $S_{i,j} = \left|\frac{\partial Y_i}{\partial P_j}\right| \cong \left|\frac{\Delta Y_i}{\Delta P_j}\right|$, where ΔP_j is the percent increment in the parameter P_j and ΔY_i the observed percent change in the output. The parameters were perturbed by 5, 10 and 15%, to assess the linearity of their impact on the output. The analysis was carried out to assess the impact of patient-specific and treatment-specific parameters on the end-of-dialysis value of several variables, and on the estimation of $\dot{V}O_{2,t}$ and $\dot{V}CO_2$ for the steady state of the model.

Parameters defining physico-chemical properties of the components of the model (i.e. dissociation constants) were not assessed because of the relative certainty of their value. The baseline values of patient and treatment parameters were set equal to those from the Sargent study for the sensitivity analysis; dialysances were set to D_{Bic} = 125 ml/min and D_{CO2} = 87 ml/min.

The outputs considered for the sensitivity analysis were end the final concentration of arterial plasma bicarbonate ($Bic_{a,f}$), the final arterial pCO_2 ($pCO_{2,a,f}$), the final tCO_2 ($tCO_{2,a,f}$), and the dose of bicarbonate administered to the patient during the session (ΔBic). The parameters tested were total hemoglobin concentration (Hb_e), oxygen saturation ($sO_{2,a}$, changed by 1% increments because of the physiological boundaries of the parameter), cardiac output ($\dot{Q}_{CO}$), net acid production rate (H_t), total plasma non-bicarbonate buffer ($tNBB_p$), tissue buffer capacity of non-bicarbonate buffer (β_{NBBt}), initial bodyweight (BW_0), initial interstitial volume ($V_{i,0}$), tissue volume (V_t), initial haematocrit (Hct_0), initial blood volume ($V_{b,0}$), ultrafiltration volume (UFV), bicarbonate dialysance (D_{bic}), CO_2 dialysance (D_{CO2}), extracorporeal blood flow ($\dot{Q}_b$), and sensitivity of tissue cells pH to external pH changes (ϕ_{pH}).

A positive sensitivity coefficient means an increase in the value of the output when the parameter was increased (Fig A1).

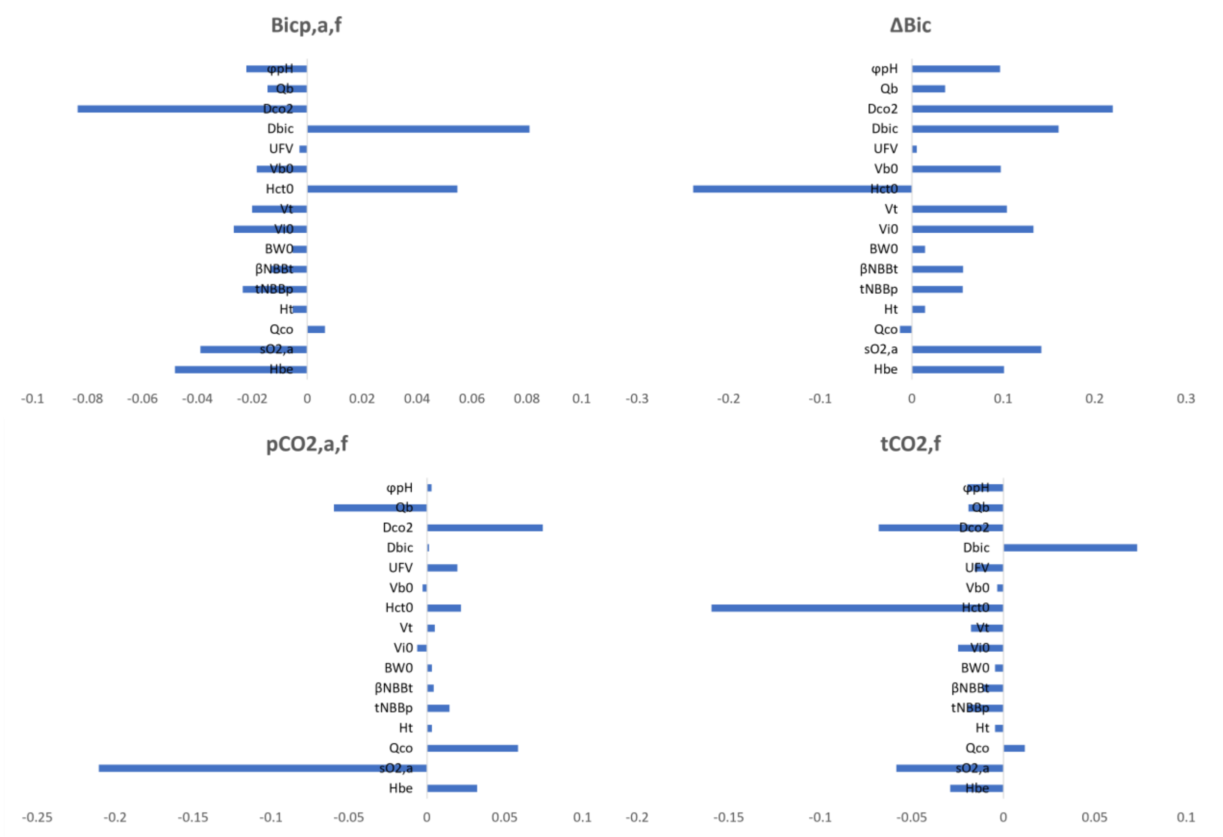

Fig A1. Sensitivity coefficients calculated for several parameters using four dialysis session outputs as reference. *The parameters were here perturbed by 10%.*

The parameters which had an effect on the identification of the steady state parameters $\dot{V}CO_{2,t}$, and $\dot{V}O_{2,t}$ were also subjected to a sensitivity analysis with said outputs (Fig A2).

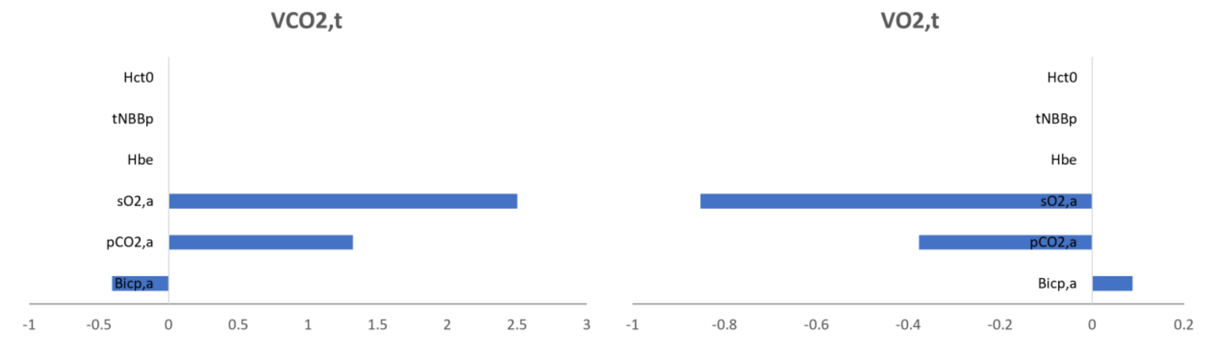

Fig A2. Sensitivity coefficients of parameters affecting the two parameters estimated for the steady-state of the model. *The parameters were here perturbed by 10%.*

The sensitivity coefficients were fairly constant in the face of an increase in the magnitude of the perturbation of the parameters (5%, 10%, 15%) with the exception of $sO_{2,a}$ (Fig A3).

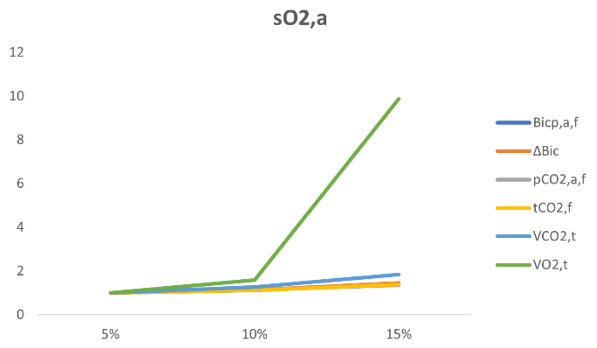

Fig A3. Sensitivity coefficients of $sO_{2,a}$ scaled on the initial value. *The coefficients for the estimated oxygen consumption rate (VO2,t) changed significantly when the perturbation was increased.*

The results of this analysis were used to direct the choice of parameters to be estimated to fit the model to the clinical data; however, the interpretation of the sensitivity indices was not straightforward because the observed outputs were variably influenced by different parameters. In general, D_{bic} and D_{CO2} were the most influential parameters on the postdialytic value of bicarbonate concentration, while $sO_{2,a}$ was the most influential on $pCO_{2,a}$. $sO_{2,a}$ was also the most influential parameter on the determination of $\dot{V}O_{2,t}$ and $\dot{V}CO_{2,t}$. Because of the strong nonlinearity of the response to changes in $sO_{2,a,}$ it was chosen to tune only D_{bic} and D_{CO2} and to assign a fixed value to $sO_{2,a}$.

Bibliography

1.Rees SE, Andreassen S. Mathematical models of oxygen and carbon dioxide storage and transport: the acid-base chemistry of blood. Crit Rev Biomed Eng. 2005;33(3):209-64. Epub 2005/04/19. doi: 10.1615/critrevbiomedeng.v33.i3.10. PubMed PMID: 15833078.

2.Siggaard-Andersen O, Wimberley PD, Göthgen I, Siggaard-Andersen M. A mathematical model of the hemoglobin-oxygen dissociation curve of human blood and of the oxygen partial pressure as a function of temperature. Clin Chem. 1984;30(10):1646-51. doi: 10.1093/clinchem/30.10.1646.

3.Siggaard-Andersen O. Acid-base biochemistry. The acid-base status of the blood. Copenhagen: Williams & Wilkins Company; 1974.

4.Andreassen S, Rees SE. Mathematical models of oxygen and carbon dioxide storage and transport: interstitial fluid and tissue stores and whole-body transport. Crit Rev Biomed Eng. 2005;33(3):265-98. Epub 2005/04/19. doi: 10.1615/critrevbiomedeng.v33.i3.20. PubMed PMID: 15833079.

5.Sargent JA, Marano M, Marano S, Gennari FJ. Acid-base homeostasis during hemodialysis: New insights into the mystery of bicarbonate disappearance during treatment. Semin Dial. 2018;31(5):468-78. Epub 2018/05/31. doi: 10.1111/sdi.12714. PubMed PMID: 29813184.

6.Park S, Paredes W, Custodio M, Goel N, Sapkota D, Bandla A, et al. Intradialytic acid-base changes and organic anion production during high versus low bicarbonate hemodialysis. Am J Physiol Renal Physiol. 2020;318(6):F1418-f29. Epub 2020/04/21. doi: 10.1152/ajprenal.00036.2020. PubMed PMID: 32308019; PubMed Central PMCID: PMCPMC7311706.
